# Alzheimer’s disease CSF biomarkers: clinical indications and rational use

**DOI:** 10.1007/s13760-017-0816-5

**Published:** 2017-07-27

**Authors:** Ellis Niemantsverdriet, Sara Valckx, Maria Bjerke, Sebastiaan Engelborghs

**Affiliations:** 1 Reference Center for Biological Markers of Dementia (BIODEM), Laboratory of Neurochemistry and Behavior, Institute Born-Bunge, University of Antwerp (UAntwerp), Antwerp, Belgium; 2 Department of Neurology and Memory Clinic, Hospital Network Antwerp (ZNA) Middelheim and Hoge Beuken, Antwerp, Belgium

**Keywords:** Alzheimer’s disease, Cerebrospinal fluid, Biomarkers, Dementia, Amyloid-β, Tau

## Abstract

This review focusses on the validation and standardization of Alzheimer’s disease (AD) cerebrospinal fluid (CSF) biomarkers, as well as on the current clinical indications and rational use of CSF biomarkers in daily clinical practice. The validated AD CSF biomarkers, Aβ_1-42_, T-tau, and P-tau_181_, have an added value in the (differential) diagnosis of AD and related disorders, including mixed pathologies, atypical presentations, and in case of ambiguous clinical dementia diagnosis. CSF biomarkers should not be routinely used in the diagnostic work-up of dementia and cannot be used to diagnose non-AD dementias. In cognitively healthy subjects, CSF biomarkers can only be applied for research purposes, e.g., to identify pre-clinical AD in the context of clinical trials with potentially disease-modifying drugs. Therefore, biomarker-based early diagnosis of AD offers great opportunities for preventive treatment development in the near future.

## Introduction

### The Alzheimer continuum: a new concept that has an impact on diagnosis, prevention, and treatment strategies

The most common type of dementia is Alzheimer’s disease (AD), affecting up to 60–70% of dementia cases and exponentially increasing in prevalence with age. AD has a long pre-clinical phase, in which neuropathological changes develop, starting at least a decade before symptom onset. At onset, complaints are insidious and mild. Subjective cognitive decline (SCD) refers to cognitive symptoms that cannot be confirmed by a neuropsychological assessment. Patients who show subtle deficits in performing daily activities in combination with objective cognitive dysfunction, confirmed by neuropsychological assessment, have reached a stage of the so-called mild cognitive impairment (MCI). If MCI is due to AD, a progressive cognitive deterioration can lead to certain functional deficits which characterize the AD dementia stage with a progression rate of 20–40% in MCI patients [1–4]. Therefore, AD is described as a continuum and includes a pre-clinical phase, SCD [5], MCI [6], and dementia due to AD [7]. In the past, the diagnosis of AD could only be suggested when the dementia stage was reached. Due to major advances in biomarker-based research, it is now possible to detect AD-related changes well before the onset of the first clinical symptoms. This provides researchers an exceptional window for early diagnosis, treatment, and prevention strategies. The cerebrospinal fluid (CSF) offers a window to the brain as the brain’s metabolism and pathology is reflected in the CSF that can easily be collected through a lumbar puncture (LP), which is a safe and well-tolerated procedure [8, 9]. This review focusses on the validation and standardization of AD CSF biomarkers, as well as on the current clinical indications and rational use of CSF biomarkers in daily clinical practice.

### The diagnosis of Alzheimer’s disease relies on different biomarker categories: amyloid-Β deposition, neurofibrillary tangles, and neuronal degeneration

The clinical diagnosis of AD is often made in accordance with the criteria from the National Institute of Neurological and Communicative Disorders and Stroke—Alzheimer’s Disease and Related Disorders Association (NINCDS-ADRDA), originating from 1984 [10]. These criteria are mainly based on excluding other systemic and brain disorders that could account for cognitive deterioration confined to the dementia stage and result at best in a diagnosis of probable AD. A clinical diagnosis of probable AD based on these criteria has been shown to achieve an average sensitivity and specificity of 81 and 70%, respectively [11]. A promising tool to increase the diagnostic accuracy of AD is the use of CSF biomarkers [7, 12–14] that can be measured in the CSF after an LP. If performed correctly, LP has a low complication rate, a high diagnostic yield, and is usually more tolerable than subjects expect [8, 9]. Biomarkers that reflect the pathology of AD already show abnormal concentrations in the pre-clinical stage of AD, thus allowing early AD diagnosis [15], even before the onset of symptoms. In 1998, a consensus report was published by the Working Group on Molecular and Biochemical Markers of Alzheimer’s Disease that determined the requirements for an ideal diagnostic biomarker for AD [16]. In general, biomarkers should be able to detect a fundamental feature of AD pathology. The value of biomarkers should also be demonstrated in neuropathologically confirmed subjects as neuropathology is still considered the gold standard for AD diagnosis. Diagnostic accuracy, sensitivity, and specificity levels should be above 80%. The test itself should be reliable and reproducible, non-invasive, simple to perform, and inexpensive [16].

CSF biomarkers are preferred over blood/plasma biochemical markers in AD to reflect brain pathophysiology, because the brain (interstitial fluid) is in direct contact with the CSF by unrestricted bi-directional flow of proteins and the CSF is secluded from direct impact of the peripheral system through the restricted transportation of molecules and proteins by the blood–CSF barrier [17]. Therefore, CSF analysis is valuable to detect markers of neurodegenerative diseases *in vivo*. CSF biomarkers are related to the three main pathological changes that occur in the AD brain (Table 1): amyloid-β (Aβ) deposition into extracellular Aβ plaques, intracellular neurofibrillary tangles (NFT) formation, and neuronal loss. The β-amyloid peptide composed of 42 amino acids (Aβ_1-42_) is the result of the cleavage of transmembrane amyloid precursor protein (APP) by β- and γ-secretases. Aβ_1-42_ is highly insoluble and aggregates into extracellular Aβ deposits in the AD brain, detected as decreased CSF Aβ_1-42_ concentrations in AD. Tau proteins are abundantly present in the cytosol of neurons, where they function to stabilize microtubules. In AD, an imbalance between kinases and phosphatases results in a hyperphosphorylation of tau, which leads to detachment of tau from microtubules and to its accumulation into NFT. During the neurodegenerative process, tau and phosphorylated tau proteins are also released into the extracellular space, resulting in increased CSF tau concentrations in AD. The formation of plaques and NFT promotes neuronal injury and, consequently, neuronal and synaptic degeneration in AD. The first Aβ plaques occur at least 10 years, and probably 20–30 years before the first symptoms [18], and are as such detectable in the CSF for early diagnosis. CSF tau biomarkers change later in the pathophysiological process compared to CSF Aβ_1-42_ [19, 20] and CSF tau is stronger correlated with cognitive decline than Aβ_1-42_ [20, 21]. CSF biomarkers give a complete overview of AD pathophysiology, and in addition, an LP is highly accessible with a low cost price, in contrast to the imaging-based markers used in AD diagnosis (Table 1). These imaging-based biomarkers include positron emission tomography (PET) with amyloid-specific probes (marker for Aβ deposition) or tau tracers (marker for neuronal injury), quantification of decreased metabolism in affected brain regions with fluorodeoxyglucose (FDG) PET imaging (marker for neuronal injury), quantification of the brain perfusion in affected brain regions with single photon emission computed tomography (SPECT) (marker for neuronal injury), and the analysis of volumetric magnetic resonance imaging (MRI) with determination of hippocampal or medial temporal lobe atrophy (marker for neuronal injury) [22–25].

**Table 1:** Overview of the different biomarkers based on the neuropathological changes in Alzheimer’s disease

Pathological change	Biomarker category	Biomarker(s)
Aβ deposition = *early marker*	Biochemical (CSF) Molecular imaging	CSF Aβ_1-42_ or Aβ_1-42_/Aβ_1-40_ PET with amyloid-specific probes
NFT formation	Biochemical (CSF)	CSF P-tau_181_
Neuronal injury = *downstream*	Biochemical (CSF) Topographical	CSF T-tau [^18^F]FDG-PET SPECT HCV / MTL atrophy on MRI

*Aβ amyloid-β*, *Aβ*_*1*-42_ β-amyloid peptide of 42 amino acids, *CSF cerebrospinal fluid*, *FDG* fluorodeoxyglucose, *HCV* hippocampal volume, *MTL* medial temporal lobe, *MRI* magnetic resonance imaging, *PET* positron emission tomography, *P-tau*_*181*_ phosphorylated tau at threonine 181, *SPECT* single photon emission computed tomography, *T-tau* total tau protein

A lot of progress has been made over the past decades to improve AD diagnosis and to validate AD CSF biomarkers in autopsy-confirmed dementia patients [26], resulting in biomarker-based research diagnostic criteria as a growing body of evidence shows that AD CSF biomarkers are reliable, reproducible, and valid as well as suitable for cut-off scores, sensitive, and specific for AD (differential) diagnosis [13]. Therefore, revised criteria of both the National Institute on Aging/Alzheimer’s Association (NIA–AA) [7, 12, 14] and the International Working Group (IWG) [13] include CSF AD biomarkers in the clinical diagnostic work-up.

#### Combining biomarkers to increase diagnostic accuracy

CSF Aβ_1-42_ (marker for Aβ deposition), total tau protein (T-tau; marker for neuronal injury), and phosphorylated tau at threonine 181 (P-tau_181_; marker for NFT) are validated and integrated CSF biomarkers in the revised diagnostic criteria of AD [7, 12–14, 27]. Changes in these three core CSF biomarkers allow diagnosing AD already in its prodromal stage [28]. Having all three biomarkers in the normal range rules out AD. Intermediate conditions require further patient follow-up [29]. The concentration of Aβ_1-42_ decreases over time in AD subjects, while P-tau_181_ and T-tau concentrations increase in AD patients compared to healthy controls (including patients with psychiatric disorders like depression) [27]. These biomarker changes almost reach their maximum in the beginning of the symptomatic phase of AD, limiting their predictive value for cognitive decline [30]. The combined use of Aβ_1–42_, T-tau, and P-tau_181_, each essential in the biomarker panel, has the highest diagnostic power to discriminate between AD and cognitively healthy controls [13, 26], with a sensitivity and specificity reaching 92 and 89%, respectively [31]. Another model based on Aβ_1-42_ and T-tau was developed that could accurately discriminate AD from controls by means of a discrimination line, which has been validated in clinical practice [32] and in autopsy-confirmed patients with sensitivity levels of 100% and specificity of 91% [26].

#### Diagnostic accuracy is independent from the analytical platform

CSF Aβ and tau proteins can be measured reliably with several analytical techniques such as single-analyte ELISAs or multi-analyte tests based on xMAP technology. Multiple studies have shown that the diagnostic accuracy of the CSF biomarker concentrations is similar when analytes are measured by means of a multi-analyte assay or single-analyte ELISA tests [33, 34]. These studies show that the clinical value of biomarkers is independent of the method by which concentrations are determined.

## The Alzheimer’s disease cerebrospinal fluid biomarker panel

### Standardization and harmonization efforts

Much effort has been put in the standardization and harmonization of AD CSF biomarker analysis and interpretation. The overall goal of all these projects is (1) to detect AD at the earliest stage possible and identify ways to track the disease through biomarkers, (2) to support advances in AD intervention, prevention, and treatment (through new diagnostic methods), and (3) to centralize data access.

The EU Joint Programme Neurodegenerative Disease Research (JPND) consortium ‘Biomarkers for AD and Parkinson’s Disease (PD)’ (BIOMARKAPD) Project (2012–2015) aimed in developing certified reference materials to harmonize assays by reducing inter-variability across international centers [35]. In this regard, the BIOMARKAPD project has united many European countries and Canada, to investigate complications related to the LP procedure in a large prospective multi-center feasibility LP study [8]. Such studies led amongst others to consensus LP guidelines to minimize risks and maximize diagnostic gain [9]. Another example of a standardization initiative is the Global Biomarkers Standardization Consortium (GBSC), with the goal to gather key researchers, clinicians, and industry, regulatory and government leaders in AD to achieve consensus on the best ways to standardize and validate biomarker tests for use in clinical practices around the world [27, 36]. In addition, the AD Neuroimaging Initiative (ADNI), the Alzheimer Biomarker Standardization Initiative (ABSI), and the JPND BIOMARKPD consortium have been launched. The Alzheimer’s Association launched an international external Quality Control (AA QC) program (2009) [36], coordinated by the Clinical Neurochemistry Laboratory in Mölndal, Sweden, and has put forth the aim to standardize CSF biomarker analyses and monitor analytical variability for Aβ and tau proteins in CSF between both research and clinical laboratories [34]. Unfortunately, the overall variability remained too high to allow assignment of universal biomarker cut-off values [36]. Further standardization of laboratory procedures and improvement of operator performance is required.

The running international standardization and harmonization efforts are very valuable as these initiatives are essential to ensure reproducible and consistent biomarker measurements, to reduce discussion in the AD field regarding biomarker values and clinical interpretation of the biomarker results, and to facilitate worldwide comparison of CSF biomarkers.

### Contributions and relevance of standardization and harmonization efforts

The overall goal of all these projects is (1) to detect AD at the earliest stage possible and identify ways to track the disease through CSF biomarkers, (2) to support advances in AD intervention, prevention, and treatment (through new diagnostic methods), and (3) to centralize data access. These initiatives have led to consensus publications on the procedure for LP/CSF sampling [9], standardization of pre-analytical factors [34, 37–39], immunoassay method validation and standardization for specific biomarkers [35, 40, 41], and the clinical use of AD CSF biomarkers [29, 42, 43].

One of the most important recommendations with a significant effect on pre-analytical variability concerns the standardization of the vial in which CSF is collected and shipped to the reference lab. It was already well known that adsorption of Aβ_1-42_ on a glassy or polystyrene vial wall causes false-low Aβ_1-42_ values. Therefore, the use of polypropylene vials is indispensable, but differences due to adsorption of Aβ_1-42_ may also occur between different brands of polypropylene vials [38, 44, 45].

Importantly, although pre-analytical and analytical variations in the concentration of CSF AD biomarkers are kept to a minimum by means of these initiatives, a recent study pointed out that simulated biomarker variability by means of shifts of ±20% in one of the three core CSF biomarkers has limited impact on the clinical accuracy of AD CSF biomarkers in MCI and autopsy-confirmed AD patients when using the IWG-2 criteria [46].

## Clinical indications for using the Alzheimer’s disease cerebrospinal fluid biomarker panel

### To increase the diagnostic accuracy in case of suspected Alzheimer’s disease

Although AD diagnostics increasingly become biomarker-based, this does not imply that every patient with suspected AD needs an LP with the exception of patients with early-onset dementia, in whom LP and CSF biomarker analysis should be routinely performed [29]. Indeed, evidence from memory clinic-based studies using autopsy-confirmed dementia patients demonstrated that the diagnostic accuracy of CSF biomarkers was comparable to the diagnostic accuracy of a clinical diagnostic work-up performed in demented patients consisting of a semi-structured interview with patient and main caregiver, general physical and clinical neurological examination, blood analysis, extensive neuropsychological examination, and brain imaging [47, 48]. However, CSF biomarkers are very valuable in those cases in which the clinical diagnostic work-up is not able to discriminate between AD and another (non-AD) type of dementia [47, 48]. Therefore, in cases with an inconclusive clinical diagnostic work-up, leading to an ambiguous AD versus non-AD differential diagnosis, the AD CSF biomarker panel has an added diagnostic value. This is as well reflected by a growing number (from 238 in 2004 to >1000 in 2016) of CSF samples that have been referred to the UAntwerp BIODEM lab, specifically for this indication [49].

### To diagnose Alzheimer’s disease in its earliest stages

#### Pre-clinical Alzheimer’s disease

The neuropathological brain lesions are present in pre-clinical AD, which is reflected by altered levels of the AD CSF biomarkers already before the onset of symptoms, and Aβ_1-42_ is the earliest marker to mirror these changes [18, 50]. This is of major importance to the health care system given the pending availability of disease-modifying drugs. Even though a CSF AD profile is much more common in patients with MCI and SCD, it is also seen in 28–36% of cognitively healthy elderly at the age of 85 [15, 50]. However, in the absence of any therapeutic consequences for pre-clinical AD, CSF biomarker analyses should not be performed in asymptomatic subjects, except in consented subjects and within the context of research and clinical trials.

#### Prodromal Alzheimer’s disease

Patients with MCI will progress to dementia at a much higher rate than healthy elderly, which makes this an excellent study population to explore the possible predictive value of CSF biomarkers [51]. In these patients, the three CSF biomarkers Aβ_1-42_, T-tau, and P-tau_181_ are strongly associated with future development of AD dementia, which was proven in a large prospective study with a mean follow-up period of more than 5 years [28] as well as in two multi-center studies [52, 53]. The AD CSF biomarkers can in fact identify those MCI patients who have prodromal AD. In the study of Hansson et al., the combination of CSF Aβ_1-42_ and T-tau at baseline yielded sensitivity and specificity levels of 95 and 83% for clinical AD diagnosis in patients with MCI [17, 28]. A high progression rate (prodromal to dementia) was confirmed by patients with a high likelihood for AD based on the NIA–AA criteria compared to patients with an intermediate or lowest likelihood for AD in a clinical setting [53].

### To discriminate Alzheimer’s disease from other neurodegenerative and cerebrovascular brain disorders: differential diagnosis

The assessment of Aβ_1-42_, T-tau, and P-tau_181_ can discriminate between AD and non-AD dementias [26, 54], but they cannot be used to confirm another type of dementia. Several other brain diseases can lead to pathological values of these core AD CSF biomarkers which might lead to possible misinterpretation of the biomarker results in the absence of clinical information. An increase in T-tau is also detected after stroke [55] and in disorders with extensive and/or rapid neuronal degeneration, such as Creutzfeldt–Jakob’s disease (CJD) [56], as opposed to other disorders with limited neuronal degeneration. For this reason, P-tau_181_ seems to be a more specific marker for AD [54]. Moreover, both Aβ_1-42_ and T-tau are detected at intermediate levels, in between normal control and abnormal AD values [26, 27, 57, 58], in non-AD patients, especially in dementia with Lewy bodies (DLB) but also in frontotemporal dementia (FTD), vascular dementia (VaD), and CJD. To improve the discriminatory power for the differential diagnosis of dementia, additional markers, more specific to the non-AD dementia can be valuable, described below (New biomarkers specific for non-AD diseases). Another problem in using CSF biomarkers for dementia diagnosis is the inter-patient variability. Indeed, heterogeneity in the amount of plaques and tangles in AD brains exist, and the plaque and tangle load in selected neocortical areas are even known to correlate with CSF biomarker concentrations, but not with Braak stages that take into account neocortical as well as allocortical brain regions [59, 60].

### To identify Alzheimer’s disease in case of suspected mixed brain pathologies

The existence of co-pathologies can lead to a potential misinterpretation of CSF biomarker results. It has been confirmed in a neuropathological study that many dementia patients have brain pathologies associated with more than one type of dementia [61]. In case of non-AD dementias, such as DLB, AD co-pathology frequently occurs [62]. Furthermore, as age is a common risk factor for neurodegenerative dementias and cerebrovascular disease (CVD), many demented patients show signs of co-pathologies on structural brain imaging at the clinical diagnostic work-up, which is confirmed by neuropathological examination [61]. It is often difficult to judge whether the vascular lesions are main contributors to the dementia, and there is a risk of over diagnosing VaD based on structural brain imaging [63]. In case of doubt between VaD or mixed AD–CVD pathology in dementia patients, the determination of CSF Aβ_1-42_, T-tau, and P-tau_181_ levels is of help to confirm or exclude the AD component in the pathophysiology of the dementia syndrome [58].

### To diagnose Alzheimer’s disease in case of a typical presentations

AD is thought to progress in a fairly stereotypical manner, as brain dysfunction begins in the hippocampal region resulting in episodic memory loss as the first and most typical symptom of AD [13]. However, there is considerable heterogeneity in the relative involvement of different cognitive domains, and therefore, the IWG-2 criteria [13], combining biomarkers and clinical phenotypes, distinguish ‘typical’ [i.e., memory-led AD) from ‘atypical’ AD. The latter comprises visual/biparietal (posterior cortical atrophy (PCA)] [64], logopenic (language), frontal (behavioral) variants of AD [65], and cerebral amyloid angiopathy (CAA) [66, 67]. Though each of these syndromes is variably associated with clinical presentations of non-AD dementias, typical and atypical AD share the same core pathology and can thus be diagnosed by the three core AD CSF biomarkers [68].

## Future cerebrospinal fluid biomarkers for Alzheimer’s disease (differential) diagnosis

### New biomarkers for differential dementia diagnosis

As mentioned before, there is an overlap in Aβ_1-42_ values between AD and non-AD dementia patients. To overcome this, other CSF biomarkers like Aβ_1-40_ are introduced to increase clinical diagnostic accuracy. Nevertheless, Aβ_1-40_ is also decreased in non-AD dementia patients, which may be explained by disease specific inter-individual variability in Aβ metabolism (high or low Aβ production and/or clearance). Therefore, the addition of the most abundant Aβ isoform, i.e., Aβ_1-40_ into an Aβ_1-42/_Aβ_1-40_ ratio, might prove to be an efficient way to diminish inter-patient variability (to control for high or low Aβ_1-42_ production, irrespective of AD pathology) [69]. Aβ_1-40_ has already been shown to improve differential dementia diagnosis in patients with intermediate P-tau_181_ levels [57]. Increased concordance between amyloid markers (amyloid-PET scan and CSF Aβ) was found in two studies when the Aβ_1-42_/Aβ_1-40_ ratio was applied compared to a CSF Aβ_1-42_ concentration alone [70, 71].

DLB and other synucleinopathies, including PD, PD dementia, and multiple system atrophy, are characterized by the accumulation of the protein α-synuclein in Lewy Bodies or glial cytoplasmic inclusions [40, 72, 73]. Although the CSF biomarkers Aβ_1-42,_ T-tau, and P-tau_181_ have an added diagnostic value for the differential dementia diagnosis, there is a need for additional markers due to often existing co-pathology in, for instance, DLB that limits the use of the AD core biomarkers for differential diagnosis [62]. As α-synuclein is the main component of Lewy bodies, it has been assessed as a biomarker for DLB [72–75]. Further research into the power of α-synuclein as a differential dementia diagnosis biomarker should clarify its potential. Furthermore, Aβ_1-37_ and Aβ_1-38_ increase the accuracy to differentiate AD from FTD or DLB [69] and also, transactive response DNA binding protein 43 (TDP-43), the main disease protein component in ubiquitin-positive, tau-, and α-synuclein-negative cytoplasmic inclusions in FTD and amyotrophic lateral sclerosis (ALS) [76]. However, TDP-43 inclusions have also been found in AD, even with a frequency of 56% in one neuropathologically confirmed AD case series [77]. Its potential as a CSF biomarker remains to be determined. Another promising new biomarker is total CSF prion protein (t-PrP), to differentiate AD from CJD. Although typical AD and CJD are clinically distinguishable, atypical AD phenotypes may present with similar features as CJD, such as very high levels of T-tau in CSF. It has been shown that t-PrP levels are lower in CJD and increased in AD compared to controls, both for clinical and neuropathological confirmed cases [78]. Furthermore, in cases where 14-3-3 protein was indicative for CJD, increased levels of t-PrP reduced the number of false positive cases amongst AD patients. T-PrP thus has the potential to increase diagnostic accuracy in atypical AD patients [78].

### Cerebospinal fluid biomarkers that predict Alzheimer’s disease progression

Although the previously described markers have a high diagnostic value, they lack the power to predict disease progression, as they only reflect the neuropathology of AD, reaching their maximum change at the MCI stage. Therefore, synaptic proteins, as markers for synaptic dysfunction/loss, are investigated as candidate markers for AD progression. The post-synaptic protein neurogranin is such a potential biomarker [79, 80]. It has been shown that neurogranin in CSF, but not plasma, was increased in AD and positively correlated with CSF tau [81]. There was a negative relationship between CSF neurogranin (and tau) and CSF Aβ_1-42_/Aβ_1-40_ [81]. De Vos and coworkers were the first to show that the CSF neurogranin/BACE1 ratio, reflecting post-synaptic/pre-synaptic integrity, is related to cognitive decline [82], emphasizing the potential of neurogranin as an AD stage marker.

## Discussion

### Early diagnosis or timely diagnosis?

Recent advances in CSF analyses and other biomarkers now enable the detection of AD in its pre-clinical phase. This fuels the debate on how and when AD should be detected, knowing that (1) effective disease-modifying drugs are currently not available and (2) there are differences in the interests and needs of individual patients (society vs research). The individual is often best served by a timely diagnosis, which could be in the MCI phase or at the ‘right’ moment for the individual, while the society may benefit from population screening when pharmacological prevention of AD is available [51]. In the absence of disease-modifying drugs, screening is debatable and more interest may be put in case finding (screening of a subgroup of the general population based on the presence of AD risk factors).

For (counseling in) clinical practice as well as for (clinical) research, CSF biomarkers are of importance to identify those MCI subjects who are not suffering from AD. For (clinical) research purposes, it is important to identify (asymptomatic) subjects who are at risk to develop AD, which is possible through Aβ biomarkers as amyloid changes are the first that occur within the AD continuum. By follow-up of at risk subjects, testing of new screening techniques could be performed, which should ultimately lead to a sensitive and non-invasive screening instrument. Indeed, a disadvantage of CSF biomarkers is that an LP is needed, which is invasive and might lead to post-LP complications. Imaging biomarkers might overcome this limitation as these are less invasive and will result in fewer post-procedure complications. At this moment, research should focus on the development/optimization of cost-efficient screening tools to be able to identify people in the asymptomatic phase once disease-modifying drugs become available. Partly due to the advance in detection techniques, research for potential disease-modifying treatments has changed its focus from the dementia phase to the MCI phase [83] and currently also to the pre-clinical phase of AD [51].

### Evidence-based clinical indications for application of the Alzheimer’s cerebrospinal fluid biomarker panel

The clinical indications to analyze CSF biomarkers are (1) neurochemical confirmation of AD in case of clinical AD (increase diagnostic accuracy, which is especially but not solely needed in case of early onset), (2) neurochemical confirmation of AD in case of doubt between AD dementia and non-AD dementia (including DLB, FTLD, VaD, and CJD), (3) neurochemical confirmation of prodromal AD in case of MCI, (4) neurochemical confirmation of AD in case of psychiatric disorders (like depression or psychosis), and (5) to rule out AD when this is clinically indicated. Over the past 10 years, the clinical indications for referral showed a shift from neurochemical confirmation of AD in case of clinical AD to differential dementia diagnosis in case of doubt between AD and non-AD dementias, prodromal AD cases, and in case of ambiguous dementia diagnosis [29, 49].

## Conclusions (Table 2)

The past decade, a lot of progress has been made with regard to standardization and harmonization of existing biomarkers for AD, dealing with pre-analytical, analytical, and post-analytical aspects. One of the most important recommendations with a significant effect on pre-analytical variability concerns the standardization of the vial in which CSF is collected and shipped to the reference lab.

**Table 2:** Recommendations for applying the core AD CSF biomarkers Aβ_1-42_, T-tau, and P-tau_181_, for clinical diagnosis

	Perform CSF analysis	New CSF biomarkers
Suspected AD diagnosis		
Early-onset dementia	Yes	
No doubt in clinical diagnosis	No	
Ambiguous clinical diagnosis	Yes	
Early AD diagnosis		
Pre-clinical AD		
Clinical research	Yes	
Cognitively healthy elderly	No	
Prodromal AD		
Clinical evidence for cognitive decline	Yes	
Differential dementia diagnosis (AD versus non-AD dementia)	Yes	Aβ_1-42_/Aβ_1-40_, t-PrP, Aβ_1-37_, Aβ_1-38_, α-synuclein
Mixed dementia pathology diagnosis		
AD as co-pathology	No	
AD versus AD–CVD	Yes	
Atypical AD diagnosis		
Diagnose atypical AD variants	Yes	

*Aβ* β-amyloid, *AD* Alzheimer’s disease, *CSF* cerebrospinal fluid, *CVD* cerebrovascular disease, *non-AD* other dementia than AD

The validated core AD CSF biomarkers have an added value in the (early, differential) diagnosis of AD and related disorders, including mixed pathologies, atypical presentations of AD, and in case of ambiguous dementia diagnosis. Analysis of the core AD CSF biomarkers is a second line diagnostic tool and should not be routinely performed in the diagnostic work-up of dementia, except in case of early-onset dementia. The AD CSF biomarker panel cannot be used to confirm clinical diagnosis of non-AD dementias, but should be used to confirm or exclude the diagnosis of AD. The AD CSF biomarkers are of great help to select subjects for clinical trials with potentially disease-modifying drugs against AD and can thus even be used to identify asymptomatic subjects who are at risk to develop symptoms of AD.

## References

[1] Han JW, Kim TH, Lee SB, Park JH, Lee JJ, Huh Y, Park JE, Jhoo JH, Lee DY, Kim KW (2012). Predictive validity and diagnostic stability of mild cognitive impairment subtypes. Alzheimer’s Dement.

[2] Koepsell TD, Monsell SE (2012). Reversion from mild cognitive impairment to normal or near-normal cognition: risk factors and prognosis. Neurology.

[3] Palmer K, Backman L, Winblad B, Fratiglioni L (2008). Mild cognitive impairment in the general population: occurrence and progression to Alzheimer disease. Am J Geriatr.

[4] Petersen RC (2016) Mild cognitive impairment. Continuum 22 (2 Dementia):404–418. doi:10.1212/CON.0000000000000313 10.1212/CON.0000000000000313 PMC5390929 27042901

[5] Jessen F, Amariglio RE, van Boxtel M, Breteler M, Ceccaldi M, Chetelat G, Dubois B, Dufouil C, Ellis KA, van der Flier WM, Glodzik L, van Harten AC, de Leon MJ, McHugh P, Mielke MM, Molinuevo JL, Mosconi L, Osorio RS, Perrotin A, Petersen RC, Rabin LA, Rami L, Reisberg B, Rentz DM, Sachdev PS, de la Sayette V, Saykin AJ, Scheltens P, Shulman MB, Slavin MJ, Sperling RA, Stewart R, Uspenskaya O, Vellas B, Visser PJ, Wagner M (2014). A conceptual framework for research on subjective cognitive decline in preclinical Alzheimer’s disease. Alzheimer’s Dement.

[6] Petersen RC (2004). Mild cognitive impairment as a diagnostic entity. J Intern Med.

[7] McKhann GM, Knopman DS, Chertkow H, Hyman BT, Jack CR, Kawas CH, Klunk WE, Koroshetz WJ, Manly JJ, Mayeux R, Mohs RC, Morris JC, Rossor MN, Scheltens P, Carrillo MC, Thies B, Weintraub S, Phelps CH (2011). The diagnosis of dementia due to Alzheimer’s disease: recommendations from the National Institute on Aging-Alzheimer’s Association workgroups on diagnostic guidelines for Alzheimer’s disease. Alzheimer’s Dement.

[8] Duits FH, Martinez-Lage P, Paquet C, Engelborghs S, Lleo A, Hausner L, Molinuevo JL, Stomrud E, Farotti L, Ramakers IHGB, Tsolaki M, Skarsgard C, Astrand R, Wallin A, Vyhnalek M, Holmber-Clausen M, Forlenza OV, Ghezzi L, Ingelsson M, Hoff EI, Roks G, de Mendonca A, Papma JM, Izagirre A, Taga M, Struyfs H, Alcolea DA, Frolich L, Balasa M, Minthon L, Twisk JWR, Persson S, Zetterberg H, van der Flier WM, Teunissen CE, Scheltens P, Blennow K (2016). Performance and complications of lumbar puncture in memory clinics: results of the multicenter lumbar puncture feasibility study. Alzheimers Dement.

[9] Engelborghs S, Niemantsverdriet E, Struyfs H, Blennow K, Brouns R, Comabella M, Dujmovic I, Van der Flier WM, Froelich L, Galimberti D, Gnanapavan S, Hemmer B, Hoff EI, Hort J, Iacobaeus E, Ingelsson M, de Jong FJ, Jonsson M, Khalil M, Kuhle J, Lleo A, De Mendonca A, Molinuevo JL, Nagels G, Paquet C, Parnetti L, Roks G, Rosa-Neto P, Scheltens P, Skarsgard C, Stomrud E, Tumani H, Visser PJ, Wallin A, Winblad B, Zetterberg H, Duits F, Teunissen C (2017). Consensus guidelines for lumbar puncture in patients with neurological diseases. Alzheimer’s Dement Diagn Assess Dis Monit.

[10] McKhann G, Drachman D, Folstein M, Katzman R, Price D, Stadlan EM (1984). Clinical diagnosis of Alzheimer’s disease: report of the NINCDS-ADRDA Work Group under the auspices of Department of Health and Human Services Task Force on Alzheimer’s Disease. Neurology.

[11] Knopman DS, DeKosky ST, Cummings JL, Chui H, Corey-Bloom J, Relkin N, Small GW, Miller B, Stevens JC (2001). Practice parameter: diagnosis of dementia (an evidence-based review). Report of the Quality Standards Subcommittee of the American Academy of Neurology. Neurology.

[12] Albert MS, DeKosky ST, Dickson D, Dubois B, Feldman HH, Fox NC, Gamst A, Holtzman DM, Jagust WJ, Petersen RC, Snyder PJ, Carrillo MC, Thies B, Phelps CH (2011). The diagnosis of mild cognitive impairment due to Alzheimer’s disease: recommendations from the National Institute on Aging-Alzheimer’s Association workgroups on diagnostic guidelines for Alzheimer’s disease. Alzheimer’s Dement.

[13] Dubois B, Feldman HH, Jacova C, Hampel H, Molinuevo JL, Blennow K, DeKosky ST, Gauthier S, Selkoe D, Bateman R, Cappa S, Crutch S, Engelborghs S, Frisoni GB, Fox NC, Galasko D, Habert MO, Jicha GA, Nordberg A, Pasquier F, Rabinovici G, Robert P, Rowe C, Salloway S, Sarazin M, Epelbaum S, de Souza LC, Vellas B, Visser PJ, Schneider L, Stern Y, Scheltens P, Cummings JL (2014). Advancing research diagnostic criteria for Alzheimer’s disease: the IWG-2 criteria. Lancet Neurol.

[14] Sperling RA, Aisen PS, Beckett LA, Bennett DA, Craft S, Fagan AM, Iwatsubo T, Jack CR, Kaye J, Montine TJ, Park DC, Reiman EM, Rowe CC, Siemers E, Stern Y, Yaffe K, Carrillo MC, Thies B, Morrison-Bogorad M, Wagster MV, Phelps CH (2011). Toward defining the preclinical stages of Alzheimer’s disease: recommendations from the National Institute on Aging-Alzheimer’s Association workgroups on diagnostic guidelines for Alzheimer’s disease. Alzheimer’s Dement.

[15] De Meyer G, Shapiro F, Vanderstichele H, Vanmechelen E, Engelborghs S, De Deyn PP, Coart E, Hansson O, Minthon L, Zetterberg H, Blennow K, Shaw L, Trojanowski JQ, AsDN Initi (2010). Diagnosis-independent Alzheimer disease biomarker signature in cognitively normal elderly people. Arch Neurol Chicago.

[16] Consensus report of the working group on:“Molecular and biochemical markers of Alzheimer’s disease”. The Ronald and Nancy Reagan Research Institute of the Alzheimer’s Association and National Institute on Aging Working Group. (1998). Neurobiology of aging 19 (2):109–116 9558143

[17] Olsson B, Lautner R, Andreasson U, Ohrfelt A, Portelius E, Bjerke M, Holtta M, Rosen C, Olsson C, Strobel G, Wu E, Dakin K, Petzold M, Blennow K, Zetterberg H (2016). CSF and blood biomarkers for the diagnosis of Alzheimer’s disease: a systematic review and meta-analysis. Lancet Neurol.

[18] Jansen WJ, Ossenkoppele R, Knol DL, Tijms BM, Scheltens P, Verhey FR, Visser PJ, Aalten P, Aarsland D, Alcolea D, Alexander M, Almdahl IS, Arnold SE, Baldeiras I, Barthel H, van Berckel BN, Bibeau K, Blennow K, Brooks DJ, van Buchem MA, Camus V, Cavedo E, Chen K, Chetelat G, Cohen AD, Drzezga A, Engelborghs S, Fagan AM, Fladby T, Fleisher AS, van der Flier WM, Ford L, Forster S, Fortea J, Foskett N, Frederiksen KS, Freund-Levi Y, Frisoni GB, Froelich L, Gabryelewicz T, Gill KD, Gkatzima O, Gomez-Tortosa E, Gordon MF, Grimmer T, Hampel H, Hausner L, Hellwig S, Herukka SK, Hildebrandt H, Ishihara L, Ivanoiu A, Jagust WJ, Johannsen P, Kandimalla R, Kapaki E, Klimkowicz-Mrowiec A, Klunk WE, Kohler S, Koglin N, Kornhuber J, Kramberger MG, Van Laere K, Landau SM, Lee DY, de Leon M, Lisetti V, Lleo A, Madsen K, Maier W, Marcusson J, Mattsson N, de Mendonca A, Meulenbroek O, Meyer PT, Mintun MA, Mok V, Molinuevo JL, Mollergard HM, Morris JC, Mroczko B, Van der Mussele S, Na DL, Newberg A, Nordberg A, Nordlund A, Novak GP, Paraskevas GP, Parnetti L, Perera G, Peters O, Popp J, Prabhakar S, Rabinovici GD, Ramakers IH, Rami L, Resende de Oliveira C, Rinne JO, Rodrigue KM, Rodriguez-Rodriguez E, Roe CM, Rot U, Rowe CC, Ruther E, Sabri O, Sanchez-Juan P, Santana I, Sarazin M, Schroder J, Schutte C, Seo SW, Soetewey F, Soininen H, Spiru L, Struyfs H, Teunissen CE, Tsolaki M, Vandenberghe R, Verbeek MM, Villemagne VL, Vos SJ, van Waalwijk, van Doorn LJ, Waldemar G, Wallin A, Wallin AK, Wiltfang J, Wolk DA, Zboch M, Zetterberg H, Amyloid Biomarker Study G (2015). Prevalence of cerebral amyloid pathology in persons without dementia: a meta-analysis. Jama.

[19] Buchhave P, Minthon L, Zetterberg H, Wallin AK, Blennow K, Hansson O (2012). Cerebrospinal fluid levels of beta-amyloid 1-42, but not of tau, are fully changed already 5 to 10 years before the onset of Alzheimer dementia. Arch Gen Psychiatry.

[20] Jack CR, Holtzman DM (2013). Biomarker modeling of Alzheimer’s disease. Neuron.

[21] Savva GM, Wharton SB, Ince PG, Forster G, Matthews FE, Brayne C, Ageing S, Medical Research Council Cognitive F (2009). Age, neuropathology, and dementia. N Engl J Med.

[22] Ottoy J, Verhaeghe J, Niemantsverdriet E, Wyffels L, Somers C, De Roeck E, Struyfs H, Soetewey F, Deleye S, Van den Bossche T, Van Mossevelde S, Ceyssens S, Versijpt J, Stroobants S, Engelborghs S, Staelens S (2017). Validation of the semi-quantitative static SUVR method for [18F]-AV45 PET by pharmacokinetic modeling with an arterial input function. J Nucl Med.

[23] Bosco P, Redolfi A, Bocchetta M, Ferrari C, Mega A, Galluzzi S, Austin M, Chincarini A, Collins DL, Duchesne S, Marechal B, Roche A, Sensi F, Wolz R, Alegret M, Assal F, Balasa M, Bastin C, Bougea A, Emek-Savas DD, Engelborghs S, Grimmer T, Grosu G, Kramberger MG, Lawlor B, Mandic Stojmenovic G, Marinescu M, Mecocci P, Molinuevo JL, Morais R, Niemantsverdriet E, Nobili F, Ntovas K, O’Dwyer S, Paraskevas GP, Pelini L, Picco A, Salmon E, Santana I, Sotolongo-Grau O, Spiru L, Stefanova E, Popovic KS, Tsolaki M, Yener GG, Zekry D, Frisoni GB (2017). The impact of automated hippocampal volumetry on diagnostic confidence in patients with suspected Alzheimer’s disease: an EADC study. Alzheimer’s Dement.

[24] Weiner MW, Veitch DP, Aisen PS, Beckett LA, Cairns NJ, Green RC, Harvey D, Jack CR, Jagust W, Morris JC, Petersen RC, Saykin AJ, Shaw LM, Toga AW, Trojanowski JQ, Alzheimer’s Disease Neuroimaging I (2017). Recent publications from the Alzheimer’s Disease Neuroimaging Initiative: reviewing progress toward improved AD clinical trials. Alzheimer’s Dement.

[25] Clark CM, Pontecorvo MJ, Beach TG, Bedell BJ, Coleman RE, Doraiswamy PM, Fleisher AS, Reiman EM, Sabbagh MN, Sadowsky CH, Schneider JA, Arora A, Carpenter AP, Flitter ML, Joshi AD, Krautkramer MJ, Lu M, Mintun MA, Skovronsky DM, Group A-AS (2012). Cerebral PET with florbetapir compared with neuropathology at autopsy for detection of neuritic amyloid-beta plaques: a prospective cohort study. Lancet Neurol.

[26] Engelborghs S, De Vreese K, Van de Casteele T, Vanderstichele H, Van Everbroeck B, Cras P, Martin JJ, Vanmechelen E, De Deyn PP (2008). Diagnostic performance of a CSF-biomarker panel in autopsy-confirmed dementia. Neurobiol Aging.

[27] Blennow K, Hampel H, Weiner M, Zetterberg H (2010). Cerebrospinal fluid and plasma biomarkers in Alzheimer disease. Nat Rev Neurol.

[28] Hansson O, Zetterberg H, Buchhave P, Londos E, Blennow K, Minthon L (2006). Association between CSF biomarkers and incipient Alzheimer’s disease in patients with mild cognitive impairment: a follow-up study. Lancet Neurol.

[29] Molinuevo JL, Blennow K, Dubois B, Engelborghs S, Lewczuk P, Perret-Liaudet A, Teunissen CE, Parnetti L (2014). The clinical use of cerebrospinal fluid biomarker testing for Alzheimer’s disease diagnosis: a consensus paper from the Alzheimer’s Biomarkers Standardization Initiative. Alzheimer’s Dement.

[30] Le Bastard N, Aerts L, Sleegers K, Martin JJ, Van Broeckhoven C, De Deyn PP, Engelborghs S (2013). Longitudinal stability of cerebrospinal fluid biomarker levels: fulfilled requirement for pharmacodynamic markers in Alzheimer’s disease. J Alzheimer’s Dis.

[31] Sunderland T, Linker G, Mirza N, Putnam KT, Friedman DL, Kimmel LH, Bergeson J, Manetti GJ, Zimmermann M, Tang B, Bartko JJ, Cohen RM (2003). Decreased beta-amyloid1-42 and increased tau levels in cerebrospinal fluid of patients with Alzheimer disease. JAMA.

[32] Andreasen N, Minthon L, Davidsson P, Vanmechelen E, Vanderstichele H, Winblad B, Blennow K (2001). Evaluation of CSF-tau and CSF-Abeta42 as diagnostic markers for Alzheimer disease in clinical practice. Arch Neurol.

[33] Le Bastard N, Coart E, Vanderstichele H, Vanmechelen E, Martin JJ, Engelborghs S (2013). Comparison of two analytical platforms for the clinical qualification of Alzheimer’s disease biomarkers in pathologically-confirmed dementia. J Alzheimers Dis.

[34] Bjerke M, Portelius E, Minthon L, Wallin A, Anckarsater H, Anckarsater R, Andreasen N, Zetterberg H, Andreasson U, Blennow K (2010) Confounding factors influencing amyloid Beta concentration in cerebrospinal fluid. International Journal of Alzheimer’s Disease 2010 10.4061/2010/986310 PMC2925386 20798852

[35] Bjerke M, Andreasson U, Kuhlmann J, Portelius E, Pannee J, Lewczuk P, Umek RM, Vanmechelen E, Vanderstichele H, Stoops E, Lewis J, Vandijck M, Kostanjevecki V, Jeromin A, Salamone SJ, Schmidt O, Matzen A, Madin K, Eichenlaub U, Bittner T, Shaw LM, Zegers I, Zetterberg H, Blennow K (2016). Assessing the commutability of reference material formats for the harmonization of amyloid-beta measurements. Clin Chem Lab Med.

[36] Mattsson N, Andreasson U, Persson S, Carrillo MC, Collins S, Chalbot S, Cutler N, Dufour-Rainfray D, Fagan AM, Heegaard NH, Robin Hsiung GY, Hyman B, Iqbal K, Kaeser SA, Lachno DR, Lleo A, Lewczuk P, Molinuevo JL, Parchi P, Regeniter A, Rissman RA, Rosenmann H, Sancesario G, Schroder J, Shaw LM, Teunissen CE, Trojanowski JQ, Vanderstichele H, Vandijck M, Verbeek MM, Zetterberg H, Blennow K, Alzheimer’s Association QCPWG (2013). CSF biomarker variability in the Alzheimer’s Association quality control program. Alzheimer’s Dement.

[37] Le Bastard N, De Deyn PP, Engelborghs S (2015). Importance and impact of pre-analytical variables on Alzheimer’s Disease biomarker levels in cerebrospinal fluid. Clin Chem.

[38] del Campo M, Mollenhauer B, Bertolotto A, Engelborghs S, Hampel H, Simonsen AH, Kapaki E, Kruse N, Le Bastard N, Lehmann S, Molinuevo JL, Parnetti L, Perret-Liaudet A, Saez-Valero J, Saka E, Urbani A, Vanmechelen E, Verbeek M, Visser PJ, Teunissen C (2012). Recommendations to standardize preanalytical confounding factors in Alzheimer’s and Parkinson’s disease cerebrospinal fluid biomarkers: an update. Biomark Med.

[39] Vanderstichele H, Bibl M, Engelborghs S, Le Bastard N, Lewczuk P, Molinuevo JL, Parnetti L, Perret-Liaudet A, Shaw LM, Teunissen C, Wouters D, Blennow K (2012). Standardization of preanalytical aspects of cerebrospinal fluid biomarker testing for Alzheimer’s disease diagnosis: a consensus paper from the Alzheimer’s Biomarkers Standardization Initiative. Alzheimer’s Dement.

[40] Andreasson U, Perret-Liaudet A, van Doorn LJCV, Blennow K, Chiasserini D, Engelborghs S, Fladby T, Genc S, Kruse N, Kuipenj HB, Kulic L, Lewczuk P, Mollenhauer B, Mroczko B, Pametti L, Vanmechelen E, Verbeek MM, Winblad B, Zetterberg H, Koel-Simmelink M, Teunissen CE (2015) A practical guide to immunoassay method validation. Front Neurol 6 10.3389/fneur.2015.00179 PMC4541289 26347708

[41] Kruse N, Persson S, Alcolea D, Bahl JM, Baldeiras I, Capello E, Chiasserini D, Bocchio Chiavetto L, Emersic A, Engelborghs S, Eren E, Fladby T, Frisoni G, Garcia-Ayllon MS, Genc S, Gkatzima O, Heegaard NH, Janeiro AM, Kovacech B, Kuiperij HB, Leitao MJ, Lleo A, Martins M, Matos M, Mollergard HM, Nobili F, Ohrfelt A, Parnetti L, de Oliveira CR, Rot U, Saez-Valero J, Struyfs H, Tanassi JT, Taylor P, Tsolaki M, Vanmechelen E, Verbeek MM, Zilka N, Blennow K, Zetterberg H, Mollenhauer B (2015). Validation of a quantitative cerebrospinal fluid alpha-synuclein assay in a European-wide interlaboratory study. Neurobiol Aging.

[42] Herukka SK, Simonsen AH, Andreasen N, Baldeiras I, Bjerke M, Blennow K, Engelborghs S, Frisoni GB, Gabryelewicz T, Galluzzi S, Handels R, Kramberger MG, Kulczynska A, Molinuevo JL, Mroczko B, Nordberg A, Oliveira CR, Otto M, Rinne JO, Rot U, Saka E, Soininen H, Struyfs H, Suardi S, Visser PJ, Winblad B, Zetterberg H, Waldemar G (2017). Recommendations for cerebrospinal fluid Alzheimer’s disease biomarkers in the diagnostic evaluation of mild cognitive impairment. Alzheimer’s Dement.

[43] Simonsen AH, Herukka SK, Andreasen N, Baldeiras I, Bjerke M, Blennow K, Engelborghs S, Frisoni GB, Gabryelewicz T, Galluzzi S, Handels R, Kramberger MG, Kulczynska A, Molinuevo JL, Mroczko B, Nordberg A, Oliveira CR, Otto M, Rinne JO, Rot U, Saka E, Soininen H, Struyfs H, Suardi S, Visser PJ, Winblad B, Zetterberg H, Waldemar G (2017). Recommendations for CSF AD biomarkers in the diagnostic evaluation of dementia. Alzheimer’s Dement.

[44] Fourier A, Portelius E, Zetterberg H, Blennow K, Quadrio I, Perret-Liaudet A (2015). Pre-analytical and analytical factors influencing Alzheimer’s disease cerebrospinal fluid biomarker variability. Clin Chim Acta Int J Clin Chem.

[45] Willemse E, van Uffelen K, Brix B, Engelborghs S, Vanderstichele H, Teunissen C (2017). How to handle adsorption of cerebrospinal fluid amyloid-beta (1-42) in laboratory practice? Identifying problematic handlings and resolving the issue by use of the Abeta42/Abeta40 ratio. Alzheimer’s Dement.

[46] Niemantsverdriet E, Goossens J, Struyfs H, Martin JJ, Goeman J, De Deyn PP, Vanderstichele H, Engelborghs S (2016). Limited diagnostic impact of CSF biomarker variability in Alzheimer’s disease. J Alzheimer’s Dis.

[47] Niemantsverdriet E, Feyen BF, Le Bastard N, Martin JJ, Goeman J, De Deyn PP, Bjerke M, Engelborghs S (Submitted, 2017) Added diagnostic value of CSF biomarkers for differential dementia diagnosis in an autopsy-confirmed cohort. Alzheimer’s Res Ther 10.3233/JAD-170927 PMC5900550 29614653

[48] Le Bastard N, Martin JJ, Vanmechelen E, Vanderstichele H, De Deyn PP, Engelborghs S (2010). Added diagnostic value of CSF biomarkers in differential dementia diagnosis. Neurobiol Aging.

[49] Somers C, Struyfs H, Goossens J, Niemantsverdriet E, Luyckx J, De Roeck N, De Roeck E, De Vil B, Cras P, Martin JJ, De Deyn PP, Bjerke M, Engelborghs S (2016). A decade of cerebrospinal fluid biomarkers for Alzheimer’s disease in Belgium. J Alzheimer’s Dis.

[50] Toledo JB, Zetterberg H, van Harten AC, Glodzik L, Martinez-Lage P, Bocchio-Chiavetto L, Rami L, Hansson O, Sperling R, Engelborghs S, Osorio RS, Vanderstichele H, Vandijck M, Hampel H, Teipl S, Moghekar A, Albert M, Hu WT, Argiles JAM, Gorostidi A, Teunissen CE, De Deyn PP, Hyman BT, Molinuevo JL, Frisoni GB, Linazasoro G, de Leon MJ, van der Flier WM, Scheltens P, Blennow K, Shaw LM, Trojanowski JQ, AsD Neuroimaging (2015). Alzheimer’s disease cerebrospinal fluid biomarker in cognitively normal subjects. Brain.

[51] De Roeck EE, Engelborghs S, Dierckx E (2016). Next generation brain health depends on early Alzheimer disease diagnosis: from a timely diagnosis to future population screening. J Am Med Dir Assoc.

[52] Mattsson N, Zetterberg H, Hansson O, Andreasen N, Parnetti L, Jonsson M, Herukka SK, van der Flier WM, Blankenstein MA, Ewers M, Rich K, Kaiser E, Verbeek M, Tsolaki M, Mulugeta E, Rosen E, Aarsland D, Visser PJ, Schroder J, Marcusson J, de Leon M, Hampel H, Scheltens P, Pirttila T, Wallin A, Jonhagen ME, Minthon L, Winblad B, Blennow K (2009). CSF biomarkers and incipient Alzheimer disease in patients with mild cognitive impairment. JAMA.

[53] Vos SJB, Verhey F, Frolich L, Kornhuber J, Wiltfang J, Maier W, Peters O, Ruther E, Nobili F, Morbelli S, Frisoni GB, Drzezga A, Didic M, van Berckel BNM, Simmons A, Soininen H, Kloszewska I, Mecocci P, Tsolaki M, Vellas B, Lovestone S, Muscio C, Herukka SK, Salmon E, Bastin C, Wallin A, Nordlund A, de Mendonca A, Silva D, Santana I, Lemos R, Engelborghs S, Van der Mussele S, Freund-Levi Y, Wallin AK, Hampel H, van der Flier W, Scheltens P, Visser PJ, AsDN Initi (2015). Prevalence and prognosis of Alzheimer’s disease at the mild cognitive impairment stage. Brain.

[54] Niemantsverdriet E, Struyfs H, Duits F, Teunissen C, Engelborghs S, Deisenhammer F, Sellebjerg F, Teunissen CE, Tumani H (2015). Techniques, contraindications and complications of CSF collection procedures. Cerebrospinal Fluid in Clinical Neurology.

[55] Hjalmarsson C, Bjerke M, Andersson B, Blennow K, Zetterberg H, Aberg ND, Olsson B, Eckerstrom C, Bokemark L, Wallin A (2014). Neuronal and glia-related biomarkers in cerebrospinal fluid of patients with acute ischemic stroke. J Cent Nerv Syst Dis.

[56] Lattanzio F, Abu-Rumeileh S, Franceschini A, Kai H, Amore G, Poggiolini I, Rossi M, Baiardi S, McGuire L, Ladogana A, Pocchiari M, Green A, Capellari S, Parchi P (2017). Prion-specific and surrogate CSF biomarkers in Creutzfeldt-Jakob disease: diagnostic accuracy in relation to molecular subtypes and analysis of neuropathological correlates of p-tau and Abeta42 levels. Acta Neuropathol.

[57] Slaets S, Le Bastard N, Martin JJ, Sleegers K, Van Broeckhoven C, De Deyn PP, Engelborghs S (2013). Cerebrospinal fluid Abeta1-40 improves differential dementia diagnosis in patients with intermediate P-tau181P levels. J Alzheimer’s Dis.

[58] Bjerke M, Zetterberg H, Edman A, Blennow K, Wallin A, Andreasson U (2011). Cerebrospinal fluid matrix metalloproteinases and tissue inhibitor of metalloproteinases in combination with subcortical and cortical biomarkers in vascular dementia and Alzheimer’s disease. J Alzheimer’s Dis.

[59] Buerger K, Ewers M, Pirttila T, Zinkowski R, Alafuzoff I, Teipel SJ, DeBernardis J, Kerkman D, McCulloch C, Soininen H, Hampel H (2006). CSF phosphorylated tau protein correlates with neocortical neurofibrillary pathology in Alzheimer’s disease. Brain.

[60] Engelborghs S, Sleegers K, Cras P, Brouwers N, Serneels S, De Leenheir E, Martin JJ, Vanmechelen E, Van Broeckhoven C, De Deyn PP (2007). No association of CSF biomarkers with APOEepsilon4, plaque and tangle burden in definite Alzheimer’s disease. Brain.

[61] De Reuck J, Deramecourt V, Cordonnier C, Leys D, Pasquier F, Maurage CA (2011). Prevalence of small cerebral bleeds in patients with a neurodegenerative dementia: a neuropathological study. J Neurol Sci.

[62] Slaets S, Le Bastard N, Theuns J, Sleegers K, Verstraeten A, De Leenheir E, Luyckx J, Martin JJ, Van Broeckhoven C, Engelborghs S (2013). Amyloid pathology influences abeta1-42 cerebrospinal fluid levels in dementia with lewy bodies. J Alzheimer’s Dis.

[63] Niemantsverdriet E, Feyen BF, Le Bastard N, Martin JJ, Goeman J, De Deyn PP, Engelborghs S (2015). Overdiagnosing vascular dementia using structural brain imaging for dementia work-up. J Alzheimer’s Dis.

[64] Crutch SJ, Lehmann M, Schott JM, Rabinovici GD, Rossor MN, Fox NC (2012). Posterior cortical atrophy. Lancet Neurol.

[65] Gorno-Tempini ML, Hillis AE, Weintraub S, Kertesz A, Mendez M, Cappa SF, Ogar JM, Rohrer JD, Black S, Boeve BF, Manes F, Dronkers NF, Vandenberghe R, Rascovsky K, Patterson K, Miller BL, Knopman DS, Hodges JR, Mesulam MM, Grossman M (2011). Classification of primary progressive aphasia and its variants. Neurology.

[66] Verbeek MM, Kremer BP, Rikkert MO, Van Domburg PH, Skehan ME, Greenberg SM (2009). Cerebrospinal fluid amyloid beta(40) is decreased in cerebral amyloid angiopathy. Ann Neurol.

[67] Renard D, Castelnovo G, Wacongne A, Le Floch A, Thouvenot E, Mas J, Gabelle A, Labauge P, Lehmann S (2012). Interest of CSF biomarker analysis in possible cerebral amyloid angiopathy cases defined by the modified Boston criteria. J Neurol.

[68] Paterson RW, Toombs J, Slattery CF, Nicholas JM, Andreasson U, Magdalinou NK, Blennow K, Warren JD, Mummery CJ, Rossor MN, Lunn MP, Crutch SJ, Fox NC, Zetterberg H, Schott JM (2015). Dissecting IWG-2 typical and atypical Alzheimer’s disease: insights from cerebrospinal fluid analysis. J Neurol.

[69] Struyfs H, Van Broeck B, Timmers M, Fransen E, Sleegers K, Van Broeckhoven C, De Deyn PP, Streffer JR, Mercken M, Engelborghs S (2015). Diagnostic accuracy of cerebrospinal fluid amyloid-beta isoforms for early and differential dementia diagnosis. J Alzheimers Dis.

[70] Niemantsverdriet E, Ottoy J, Somers C, De Roeck E, Struyfs H, Soetewey F, Verhaeghe J, Van den Bosche T, Van Mossevelde S, Goeman J, De Deyn PP, Marien P, Versijpt J, Sleegers K, Van Broeckhoven C, wyffels L, Albert A, Ceyssens S, Stroobants S, Staelens S, Bjerke M, Engelborghs S (Submitted, 2017) The CSF Aβ1-42/Aβ1-40 ratio improves concordance with amyloid-PET for diagnosing Alzheimer’s disease in a clinical setting J Alzheimer’s Dis 10.3233/JAD-170327 PMC5611891 28869470

[71] Leuzy A, Chiotis K, Hasselbalch SG, Rinne JO, de Mendonca A, Otto M, Lleo A, Castelo-Branco M, Santana I, Johansson J, Anderl-Straub S, von Arnim CA, Beer A, Blesa R, Fortea J, Herukka SK, Portelius E, Pannee J, Zetterberg H, Blennow K, Nordberg A (2016). Pittsburgh compound B imaging and cerebrospinal fluid amyloid-beta in a multicentre European memory clinic study. Brain.

[72] Simonsen AH, Kuiperij B, El-Agnaf OM, Engelborghs S, Herukka SK, Parnetti L, Rektorova I, Vanmechelen E, Kapaki E, Verbeek M, Mollenhauer B (2016). The utility of alpha-synuclein as biofluid marker in neurodegenerative diseases: a systematic review of the literature. Biomark Med.

[73] Slaets S, Vanmechelen E, Le Bastard N, Decraemer H, Vandijck M, Martin JJ, De Deyn PP, Engelborghs S (2014). Increased CSF alpha-synuclein levels in Alzheimer’s disease: correlation with tau levels. Alzheimer’s Dement.

[74] Mollenhauer B, Locascio JJ, Schulz-Schaeffer W, Sixel-Doring F, Trenkwalder C, Schlossmacher MG (2011). alpha-Synuclein and tau concentrations in cerebrospinal fluid of patients presenting with parkinsonism: a cohort study. Lancet Neurol.

[75] Parnetti L, Chiasserini D, Bellomo G, Giannandrea D, De Carlo C, Qureshi MM, Ardah MT, Varghese S, Bonanni L, Borroni B, Tambasco N, Eusebi P, Rossi A, Onofrj M, Padovani A, Calabresi P, El-Agnaf O (2011). Cerebrospinal fluid Tau/alpha-synuclein ratio in Parkinson’s disease and degenerative dementias. Mov Disord.

[76] Neumann M, Sampathu DM, Kwong LK, Truax AC, Micsenyi MC, Chou TT, Bruce J, Schuck T, Grossman M, Clark CM, McCluskey LF, Miller BL, Masliah E, Mackenzie IR, Feldman H, Feiden W, Kretzschmar HA, Trojanowski JQ, Lee VM (2006). Ubiquitinated TDP-43 in frontotemporal lobar degeneration and amyotrophic lateral sclerosis. Science.

[77] Arai T, Mackenzie IR, Hasegawa M, Nonoka T, Niizato K, Tsuchiya K, Iritani S, Onaya M, Akiyama H (2009). Phosphorylated TDP-43 in Alzheimer’s disease and dementia with Lewy bodies. Acta Neuropathol.

[78] Dorey A, Tholance Y, Vighetto A, Perret-Liaudet A, Lachman I, Krolak-Salmon P, Wagner U, Struyfs H, De Deyn PP, El-Moualij B, Zorzi W, Meyronet D, Streichenberger N, Engelborghs S, Kovacs GG, Quadrio I (2015). Association of cerebrospinal fluid prion protein levels and the distinction between Alzheimer disease and Creutzfeldt-Jakob disease. JAMA Neurol.

[79] Thorsell A, Bjerke M, Gobom J, Brunhage E, Vanmechelen E, Andreasen N, Hansson O, Minthon L, Zetterberg H, Blennow K (2010). Neurogranin in cerebrospinal fluid as a marker of synaptic degeneration in Alzheimer’s disease. Brain Res.

[80] Tarawneh R, D’Angelo G, Crimmins D, Herries E, Griest T, Fagan AM, Zipfel GJ, Ladenson JH, Morris JC, Holtzman DM (2016). Diagnostic and prognostic utility of the synaptic marker neurogranin in Alzheimer disease. JAMA Neurol.

[81] De Vos A, Jacobs D, Struyfs H, Fransen E, Andersson K, Portelius E, Andreasson U, De Surgeloose D, Hernalsteen D, Sleegers K, Robberecht C, Van Broeckhoven C, Zetterberg H, Blennow K, Engelborghs S, Vanmechelen E (2015). C-terminal neurogranin is increased in cerebrospinal fluid but unchanged in plasma in Alzheimer’s disease. Alzheimers Dement.

[82] De Vos A, Struyfs H, Jacobs D, Fransen E, Klewansky T, De Roeck E, Robberecht C, Van Broeckhoven C, Duyckaerts C, Engelborghs S, Vanmechelen E (2016) The CSF neurogranin/BACE1 ratio is a potential correlate of cognitive decline in Alzheimer’s Disease. J Alzheimer’s Dis 10.3233/JAD-160227 PMC4981899 27392859

[83] Sevigny J, Chiao P, Bussiere T, Weinreb PH, Williams L, Maier M, Dunstan R, Salloway S, Chen T, Ling Y, O’Gorman J, Qian F, Arastu M, Li M, Chollate S, Brennan MS, Quintero-Monzon O, Scannevin RH, Arnold HM, Engber T, Rhodes K, Ferrero J, Hang Y, Mikulskis A, Grimm J, Hock C, Nitsch RM, Sandrock A (2016). The antibody aducanumab reduces Abeta plaques in Alzheimer’s disease. Nature.

